# Needs assessment of Wisconsin primary care residents and faculty regarding interest in global health training

**DOI:** 10.1186/1472-6920-9-36

**Published:** 2009-06-24

**Authors:** Terese A Bauer, James Sanders

**Affiliations:** 1 Family Medicine Department, Aurora-University of Wisconsin Medical Group, Milwaukee, Wisconsin, USA; 2 Family Medicine Department, Medical College of Wisconsin, Milwaukee, Wisconsin, USA

## Abstract

**Background:**

The primary objectives of this study were to assess Wisconsin's primary care residents' attitudes toward international health training, the interest among faculty to provide IH training, and the preferred modality of IH training.

**Methods:**

Surveys were administered using 505 residents and 413 medical faculty in primary care residencies in Wisconsin. Results from 128 residents and 118 medical school faculty members were collected during the spring of 2007 and analyzed.

**Results:**

In total, 25% of residents (128/505) and 28% of faculty (118/413) responded to the survey. A majority of residents (58%) and faculty (63%) were interested in global health issues. Among residents, 63% planned on spending professional time working abroad. Few residents (9%) and faculty (11%) assess their residencies as preparing residents well to address topics relating to international health. The survey indicates that adequate faculty in Wisconsin could provide mentorship in international health as 47% (55) of faculty had experience working as a physician internationally, 49% (58) of faculty spend more than 25% clinical time caring for patient from underserved communities and 39% (46) would be willing to be involved with developing curriculum, lecturing and/or mentoring residents in international health.

**Conclusion:**

Overall, the majority of the respondents expressed high interest in IH and few felt prepared to address IH issues indicating a need for increased training in this area. The findings of this survey are likely relevant as a prototype for other primary care residencies.

## Background

As the world grows more connected and people cross biozones with increasing ease, issues of global health and worldwide equitable health care become more pressing [1-15]. For physicians working within the US, increasingly pertinent international health (IH) topics include international adoptions, returning travelers, immigrant/migrant health, infectious disease, the equity of global health systems, international medical volunteering, etc [1]. Using infectious disease as one example of this trend, imported global disease outbreaks pose profound challenges to the US health system. Multi-drug resistant TB, HIV, SARS, the prospect of Avian Flu, and the recent pandemic of H1N1 Influenza all underpin this ongoing threat.

The trend of cross border migration of both people and disease is likely to continue, if not accelerate. All health systems, Wisconsin's being no exception, would do well to have physicians who are learned in international disease trends, the clinical manifestations of common global infectious diseases, and the distribution of limited health resources.

To some degree, international health (IH) training is already happening on a national level. Driven by both a push from below by an increasing student interest [3,4] and a pull from the top from administrators recognizing the value of offering IH curricula [5,6], there has been steady advancement in IH curriculum development. The benefits attributed to international health electives are extensive: positive influence on clinical diagnostic and communication skills; attitudinal changes regarding public health, health service delivery, cross-cultural communications, and challenges of health care in underserved communities; increased resource consciousness; increased knowledge in tropical disease; and an association with choosing a career in primary care and underserved communities [7,8].

The Global Health Education Consortium, which counts more than 70 medical schools amongst its members, does offer guidance for the development of global health curriculum within medical schools [1,9]. However, at the post-graduate residency level, there is little coordination. Currently, IH curriculums are being implemented in pediatrics [10,11], internal medicine [12], family medicine [13,14], surgery [15], and emergency medicine [16] residency training programs. The quality of these IH training programs varies considerably [17].

In order to assess the need for IH training in Wisconsin, the investigators conducted two web-based surveys. The primary objectives of these surveys were to assess Wisconsin's primary care residents' attitudes toward international health training, the interest among faculty to provide IH training, and the preferred modality of IH training.

## Methods

A cross-sectional web-based survey was conducted using all primary care faculty and residents in Milwaukee and Madison, Wisconsin as a sample of convenience. Primary care includes Internal Medicine, Pediatrics, Medicine-Pediatrics and Family Medicine. Two survey instruments were developed; one for residents and one for faculty physicians. Questions were structured as answerable with yes/no, open ended, rank list, or used a 5-point Likert-type scale. Prior to being administered, both surveys were reviewed, edited, and validated by several faculty. See additional files 1 and 2 for complete survey questions.

IRB approval was gained from all participating institutions. A web-based software application available from the University of Wisconsin was used to facilitate delivery and collection of the survey. During the spring of 2007, a survey was emailed to all Internal Medicine, Pediatric, Medicine-Pediatric, and Family Medicine resident physicians and faculty physicians at the Medical College of Wisconsin (MCW) and the University of Wisconsin's Milwaukee and Madison campuses. The initial survey request was followed up with two email reminders each one week apart.

In order to identify interest in IH, residents and faculty were asked to rank their own interest on a Likert scale, indicate their individual international health experiences, and indicate their level of commitment to future IH activities. In order to assess the need for a structured IH curriculum, residents and faculty were asked to rank their residency programs on a 5-point Likert scale regarding current preparedness to address IH topics as well as their programs' ability to train physicians to work internationally or domestically with underserved communities.

Data was analyzed descriptively. Chi squared with Yates' correction or Fisher's exact test was used to determine statistical significance. Multivariate binary logistic regression was used to identify independent predictor variables. Likert-type questions were analyzed combining the responses 4 and 5 to indicate a positive response. All responses from residents were analyzed geographically, institutionally, by year of residency, and by individual specialties. All responses from faculty were analyzed geographically, institutionally, by individual specialties, and by those having a Masters of Public Health. Statistically significant differences (p < 0.05) are reported.

## Results

In total, 25% of residents (128/505) and 28% of faculty (118/413) responded to the survey. Of note, published data on the response rate to web-based surveys shows the expected response rate is 20.7% [18]. Resident respondents included 45 Internal Medicine residents, 40 Pediatric residents, 41 Family Medicine residents, and 2 Internal Medicine-Pediatric residents with a wide distribution of year in training (40 PGY-1, 42 PGY-2, 42 PGY-3 and 4 PGY-4 respondents). Ninety seven percent of residents surveyed were in a 3-year primary care residency. There were 5 International Medical Graduates (IMG) who participated in the survey. Faculty respondents included 45 Internal Medicine faculty, 27 Pediatric faculty, 42 Family Medicine faculty, and 4 Internal Medicine-Pediatric faculty. Among both resident and faculty respondents there was adequate representation from all query sites.

### Past experiences

Thirty-four residents (26%) stated that they had participated in IH electives during medical school. Most of these IH electives lasted from 3 weeks to 4 months (70%).

Eleven residents (8%) responded to having participated in an IH elective during their residency. These residents visited 11 countries mostly staying for 3 weeks to 4 months (77%). Seven of these residents reported having also taken an IH elective during medical school. One resident chose to return to the same country during both medical school and residency. Six residents were from UW's Milwaukee campus, five residents were from MCW, and one resident was from UW's Madison campus.

When faculty were asked, "Have you had experience working as a physician internationally, including providing medical care, community medicine, teaching, or public health activities?," 47% (55) report having had at least one IH experience. Additionally, 49% (58) of faculty reported spending more than 25% of their clinical time caring for patients from underserved communities.

Among faculty, 16% (19) have a Master's in Public Health of whom four had special focus on IH issues. Specifically, one faculty member has a Masters in Epidemiology and PhD in Anthropology, one faculty focused on addiction, HIV prevention, and tuberculosis, another faculty focused on International Public Health, and one faculty focused on Tropical Medicine.

### Attitudes

**Figure 1:**
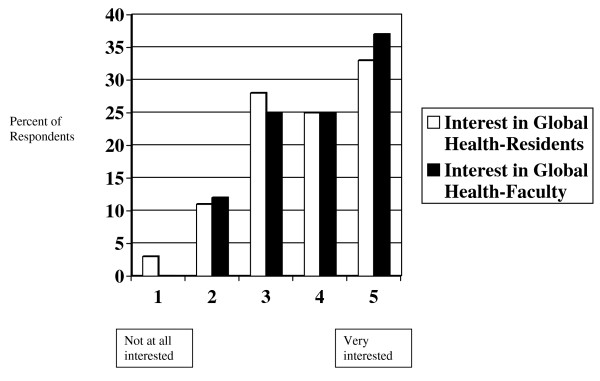
How interested are you in learning about global health issues during your residency?

A majority of residents (58%) and faculty (63%) responded positively to the question "How interested are you in learning about global health issues during your residency?" (See figure 1) There was more interest from faculty in the University of Wisconsin system (p = 0.04).

Of the residents that had taken an IH elective rotation during their residency, 82% expressed commitment to continue working in IH and 100% expressed commitment to work with the poor and underserved domestically.

Sixty three percent of the resident respondents planned on spending professional time working abroad. Most of these (70%) stated interest on spending <20% of their professional time working abroad. Independent predictor variables for residents planning on spending professional time in international health were having participated in an international elective during medical school (p < 0.01) and commitment to work with poor and underserved communities (p < 0.01).

Thirty nine percent of responding faculty (46) stated that they would be willing to be involved with developing curriculum, lecturing and/or mentoring residents in IH.

Independent predictor variables for being willing to be involved with developing curriculum, lecturing, and/or mentoring residents in IH include interest in IH (p < 0.01) and experience in IH (p = 0.014). Further, a predictor of faculty interest in IH was spending significant amount of clinic time (>25%) caring for patients from underserved communities (p = 0.01).

**Figure 2:**
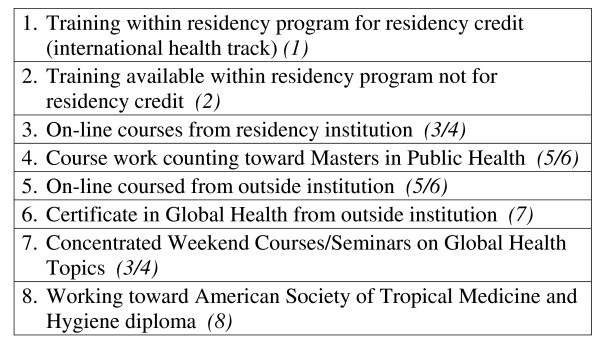
**Please rank the following medical training programs in order of your interest**. Residents (*Faculty*).

**Figure 3:**
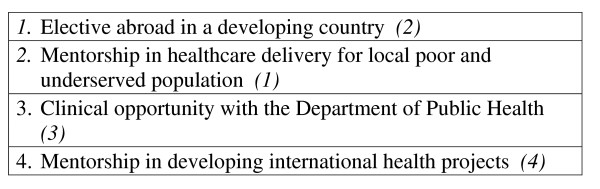
**Please rank the following clinical experience in order of importance**. Residents (*Faculty*).

**Figure 4:**
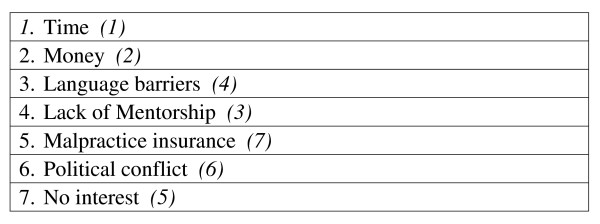
**Rank the barriers that prevent you from pursuing international health**. Residents (*Faculty*).

Residents and faculty were asked to rank medical training programs, clinical experiences, and barriers in order of importance as they pertain to IH training (see figures 2, 3, 4). In addition to the barriers in the rank list, residents and faculty cited the following barriers: family medicine outpatient requirements from the ACGME; available, trustworthy international sites for clinical experiences; family commitments; restrictions on funding of residents; and a limit of 1 month elective abroad.

When asked if there were any additional comments or questions, 4 residents and 5 faculty emphasized that they would be interested in helping develop IH curriculum, 1 resident and 3 faculty expressed that IH training is a bad idea, and 6 residents and 3 faculty elaborated on additional barriers to IH training.

### Influences

Thirty-eight residents described 41 motivating influences for seeking IH experiences. Strong desire, internal motivation, parental influence or being an IMG was cited by 19 residents. A previous IH experience or availability of an IH opportunity was cited by 14 residents. There were 5 residents motivated by concern for healthcare disparity or sense of social responsibility while 3 residents cited mentorship as an influencing factor.

### Perceptions on current training

**Figure 5:**
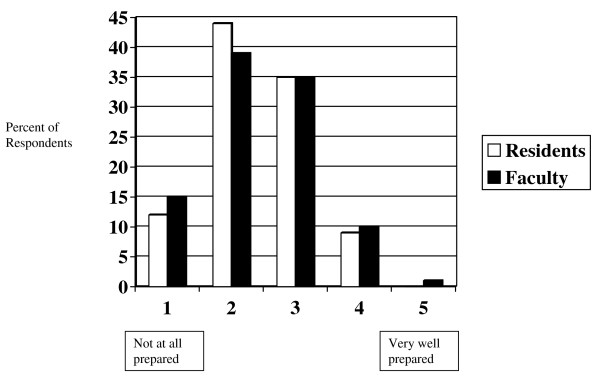
**Residency preparedness: address international health topics**.

Few residents (9%) and faculty (11%) assess their residencies as preparing residents well to address topics relating to IH (see figure 5). Few residents (10%) and faculty (8%) assess their residencies as preparing residents well to work internationally. However, most residents (53%) and faculty (50%) assess their residencies as preparing residents to work with poor and underserved communities. Overall, there is an increased sense of preparedness for both IH electives and working with the poor by family medicine residents (p = 0.001) and faculty (p = 0.05) when compared with their counterparts in internal medicine and pediatrics combined.

### Curriculum

In terms of modality of training for IH topics, 86% (109) of residents and 70% (83) of faculty were interested in incorporating IH curriculum into general lecture series while 48% (61) of residents and 45% (54) of faculty were interested in participating in a quarterly global health interest group. Interestingly, when divided by year of residency, there was no significant difference in the desire to incorporate IH curriculum into their residency's general lecture series; however, there was more of an interest among PGY-1's to become involved with a quarterly meeting (p < 0.01).

## Discussion

### Expressed need

The majority of surveyed primary care residents and faculty identify an interest in international health. While a majority of residents were interested in IH issues, few felt prepared to address these issues. This finding held up across specialties, residency location, and year of training. The survey showed that a large number of faculty and residents have pursued IH experiences and want to pursue more. This finding suggests two points for further consideration. First, there is a need for improved integration of IH curriculum across institutions and primary care specialties within Wisconsin. Secondly, the findings could serve as a baseline and be extrapolated to other locations within the US.

### Adequate resources

The survey indicates that ample faculty in Wisconsin could provide mentorship in international health. Many faculty physicians in the primary care residencies have experience working as a physician internationally. Half of faculty physicians spend more than 25% clinical time caring for patient from underserved communities. Pertinent competencies that are shared between underserved domestic populations and IH populations include: cultural awareness, use of scarce resources, social determinants of health, health disparities, human rights, advocacy, language skills, literacy, and community medicine. Additionally, despite being engaged in their current faculty responsibilities, there is a substantial cohort of physicians who would be willing to be involved with developing curriculum, lecturing and/or mentoring residents in international health.

### Curriculum development

One criticism in expanding IH learning opportunities during residency has been that a residency program has little if any influence upon a physician's choice to pursue future IH opportunities. The survey suggests evidence to the contrary when analyzing the question regarding the critical moment that led to the pursuit of an IH elective. Of the respondents to this question, 41% sited a factor that could be influenced by exposure to IH training during residency. Specifically, mentorship in IH, availability of an IH opportunity, and having an IH experience are factors that can be provided by a primary care residency program. Further study will be needed to better understand the nature of effective mentorship and opportunities within an IH context.

Most residents plan on spending some professional time working abroad in international health with the majority committing less than or equal to 10%. Thus, residents would be best served if developers of IH curriculum focus on skills needed for this type of professional effort.

Given the rapid pace that most residencies place upon the learner, the results of this survey indicate that residents and faculty would prefer to integrate IH curriculum into general lecture series rather than utilizing external IH training resources (see figure 2). Another option is to combine local resources to organize a quarterly global health interest group. The survey showed that nearly half of residents and faculty have interest to participate in this type of opportunity. Gatherings like this are useful for the sharing of experiences and ideas, learning from one another's work, attracting speakers of merit, participating in journal clubs, etc.

### Funding

An interesting finding of the survey was that at the UW-Madison site only 1 resident had participated in an IH elective. This striking discrepancy between UW – Madison and the other two sites is significant. As noted in the comment section of UW-Madison resident surveys, this discrepancy is due to external constraints placed upon the training programs at UW-Madison and not due to any inherent demographic or educational differences. UW-Madison is a training institution that derives much of its budget from state tax revenues. And while many residency programs must forego Medicare flow through monies when a resident leaves campus for an away elective, public institutions, such as UW-Madison, are even more acutely accountable to show that those being paid from the state tax rolls are serving the needs of the state. This barrier to IH involvement is certainly not unique to UW-Madison and may be shared by most state-sponsored training sites across the country. Clearly, in order to effectively transform the curriculum at all Wisconsin's primary care residencies, funding streams need to be secured. State legislators must be educated about the importance of IH curriculum within the overall graduate medical education context. Two strategies would be to make the explicit linkage to work force development and the increased risk that citizens around the state have regarding international disease processes.

### Impact on local health care

Incorporating IH curriculum into the general lecture primary care residency lecture series would impact health care close to home. As John Frey, M.D, editor of the Wisconsin Medical Journal, stated, "In the Wisconsin County Health Rankings, developed by the University of Wisconsin Population Health Institute, we find the same factors that affect prenatal and neonatal risk in the developing world affecting the citizens of the state [19]." This survey indicates that there is a correlation between commitment to working in IH and commitment to serving the underserved. An obvious place to start IH curricular development would be with the training modules and tools already developed for teaching primary care residents how to care for under-served populations.

Further, the health system needs to be responsive to the increasing likelihood that many of our state's residents will return from or immigrate from foreign lands with diseases that are uncommon in our communities [20]. Providing IH education including common clinical presentation of tropical disease would improve the diagnosis and treatment of the returned international traveler and immigrant populations. Looking forward, as the state's epidemiological surveillance and monitoring become more sophisticated and take advantage of web-based technologies, having a work force that is already literate with tropical disease processes and their presentation will accelerate the diffusion of important medical information and achieve higher levels of appropriate response.

### Limitations

Limitations to this study include volunteer bias, small sample size, recall bias, and lack of respondents' demographic information. While the small sample size was adequate to detect statistical significant differences among respondents of the survey, a larger sample size would have been preferred. Taken together, these limitations decrease the ability to generalize the results of the survey.

## Conclusion

This needs assessment survey of Wisconsin primary care residents and faculty shows an interest in and a need for IH training in Wisconsin. Wisconsin's primary care residents have an expressed interest in IH as well as commitment to spending professional time in IH. Policy makers need to take note of the importance of IH curriculum and address the funding discrepancies that limit IH training at state sponsored training centers. Ultimately, IH training will allow our state to be proactive rather than reactive in the face of global health changes and an increasingly connected world.

Wisconsin is a state that has no international borders and limited international flights. Yet the facts show that the public health of Wisconsin's citizens is affected by global health issues and Wisconsin's primary care residents express a need for IH training. The findings of this survey are likely relevant as a prototype for primary care residencies around the country.

## Competing interests

Both authors are involved with global health training of residents at their institutions. The authors declare that they have no financial competing interests.

## Authors' contributions

TAB designed and carried out the survey. TAB analyzed the data of the survey and drafted the manuscript. JS reviewed, edited, and validated the survey. Both authors read, edited, and approved the final manuscript.

## Pre-publication history

The pre-publication history for this paper can be accessed here:



## Supplementary Material

Additional file 1 **Survey Questions, Residents**. The survey tool sent to Wisconsin primary care residents to assess the need for international health training. Click here for file

Additional file 2 **Survey Questions, Faculty**. The survey tool sent to Wisconsin primary care faculty to assess the need for international health training. Click here for file
