# Supplementary material for: Needs assessment of Wisconsin primary care residents and faculty regarding interest in global health training
Source: BMC Med Educ. 2009 Jun 24;9:36. doi: 10.1186/1472-6920-9-36 (PMC2713225; doi:10.1186/1472-6920-9-36)
Supplement: Additional file 1 — Survey Questions, Residents. The survey tool sent to Wisconsin primary care residents to assess the need for international health training. [file 1472-6920-9-36-S1.doc]

Need for International Health Training-Residents

**Q1** What is your residency program?

1. MCW Internal Medicine Residency
2. MCW Pediatric Residency
3. MCW Internal Medicine-Pediatric Residency
4. Columbia St. Mary’s Family Medicine Residency
5. St. Joseph Family Medicine Residency
6. Racine Family Practice Residency Program
7. Waukesha Family Practice Residency
8. Aurora-UW St. Luke’s Family Medicine Residency
9. Aurora-UW Internal Medicine Residency
10. UW Internal Medicine Residency
11. UW Pediatric Residency
12. UW Family Medicine Residency
13. Surgical Residency

**Q2** What year are you in training?

1. PGY-1
2. PGY-2
3. PGY-3
4. PGY-4
5. PGY-5

**Q3** How interested are you in learning about global health issues during your residency? (1 being “not at all interested” and 5 being “very interested”)

1 2 3 4 5

**Q4** Have you participated in international elective during medical school?

1. Yes
2. No

**Q5** If so where have you gone? For how long? What did you do?

**Q6** Have you participated in international electives during residency?

1. Yes
2. No

**Q7** If so where have you gone? For how long? What did you do?

**Q8** If you have had experience in international health, what was the critical moment or incident that led to your pursuit of this experience?

**Q9** How well has your residency curriculum prepared you to address topics relating to international health including preparing a patient for international travel, assessing the returned traveler, international adoption, and immigrant health care? (1 being “not at all” and 5 being “very well prepared”)

1 2 3 4 5

**Q10** When you are finished with residency, do you plan on spending any professional time working abroad in international health>?

1. Yes
2. No

**Q11** If yes, how much time?

1. < or = 10%
2. 11-20%
3. 21-40%
4. 41-60%
5. 61-80%
6. >80%

**Q12** How well has your residency program prepared you to work internationally? (1 being “not at all” and 5 being “very well prepared”)

1 2 3 4 5

**Q13** When you are finished with residency, do you plan on spending any professional time working with poor and underserved communities (whether home or abroad)?

1. Yes
2. No

**Q14** If yes, how much time?

1. < or = 10%
2. 11-20%
3. 21-40%
4. 41-60%
5. 61-80%
6. >80%

**Q15** How well has your residency program prepared you to work with poor and underserved communities? (1 being “not at all” and 5 being “very well prepared”)

1 2 3 4 5

**Q16** Would you be interested in incorporating international health curriculum into your general lecture series?

1. Yes
2. No

**Q17** Would you be interested in participating in quarterly Global Health Interest Group?

1. Yes
2. No

**Q18** Please rank the following med training programs in order of your interest.

1. Training available within residency program not for residency credit (global health lectures, electives abroad)
2. Training within residency program for residency credit (international health track)
3. On-line courses from residency institution
4. On-line courses from outside institution
5. Certificate in Global Health from outside institution
6. Concentrated Weekend Courses/Seminars on Global Health topics
7. Course work counting toward Masters in Public Health
8. Working toward American Society of Tropical Medicine and Hygiene diploma (Nationally recognized board certification)

**Q19** Please rank the following clinical experience in order of importance.

1. Elective abroad in a developing country
2. Mentorship in developing internatio0nal health projects
3. Mentorship in healthcare delivery for local poor and underserved populations
4. Clinical opportunity with the Department of Public Health

**Q20** Please rank the barriers that prevent you from pursuing international health.

1. Time
2. Money
3. Malpractice Insurance
4. Language barriers
5. Political conflict
6. Lack of Mentorship
7. No Interest

**Q21** Any additional comments or questions?
