# Supplementary material for: Needs assessment of Wisconsin primary care residents and faculty regarding interest in global health training
Source: BMC Med Educ. 2009 Jun 24;9:36. doi: 10.1186/1472-6920-9-36 (PMC2713225; doi:10.1186/1472-6920-9-36)
Supplement: Additional file 2 — Survey Questions, Faculty. The survey tool sent to Wisconsin primary care faculty to assess the need for international health training. [file 1472-6920-9-36-S2.doc]

Need for International Health Training-Faculty

**Q1** What is your residency program?

1. MCW Internal Medicine Residency
2. MCW Pediatric Residency
3. MCW Internal Medicine-Pediatric Residency
4. Columbia St. Mary’s Family Medicine Residency
5. St. Joseph Family Medicine Residency
6. Racine Family Practice Residency Program
7. Waukesha Family Practice Residency
8. Aurora-UW St. Luke’s Family Medicine Residency
9. Aurora-UW Internal Medicine Residency
10. UW Internal Medicine Residency
11. UW Pediatric Residency
12. UW Family Medicine Residency
13. Surgical Residency

**Q2** How interested are you in global health issues? (1 being “not at all interested” and 5 being “very interested”)

1 2 3 4 5

**Q3** Have you had experience working as a physician internationally, including providing medical care, community medicine, teaching, or public health activities?

1. Yes
2. No

**Q4** If yes, where have you gone? For how long? What did you do?

**Q5** Do you have a Masters in Public Health?

1. Yes
2. No

**Q6** If yes, did you focus on global health issues or underserved communities?

**Q7** If yes what area?

**Q8** Doy ou spend a significant amount of your clinic time (>25%) caring for patients from underserved communities?

1. Yes
2. No

**Q9** If yes, people from which communities?

1. urban African American
2. Hispanic
3. Hmong
4. Recent Immigrant
5. Other, please specify

**Q10** How well does your residency program prepare residents to address topics relating to international health including preparing a patient for international travel, assessing the returned traveler, international adoption, and immigrant health care? (1 being “not at all” and 5 being “very well prepared”)

1 2 3 4 5

**Q11** How well does your residency program prepare residents to work internationally? (1 being “not at all” and 5 being “very well prepared”)

1 2 3 4 5

**Q12** How well does your residency program prepare residents to work with poor and underserved communities? (1 being “not at all” and 5 being “very well prepared”)

1 2 3 4 5

**Q13** Would you be interested in incorporating international health curriculum into your required curriculum?

1. Yes
2. No

**Q14** Would you be willing to be involved with developing curriculum, lecturing, and/or mentoring residents in international health?

1. Yes
2. No

**Q15** Would you be interested in participating in quarterly Global Health Interest Group?

1. Yes
2. No

**Q16** Please rank the following med training programs in order of importance.

1. Training available within residency program not for residency credit (global health lectures, electives abroad)
2. Training within residency program for residency credit (international health track)
3. On-line courses from residency institution
4. On-line courses from outside institution
5. Certificate in Global Health from outside institution
6. Concentrated Weekend Courses/Seminars on Global Health topics
7. Course work counting toward Masters in Public Health
8. Working toward American Society of Tropical Medicine and Hygiene diploma (Nationally recognized board certification)

**Q17** Please rank the following clinical experience in order of importance.

1. Elective abroad in a developing country
2. Mentorship in developing internatio0nal health projects
3. Mentorship in healthcare delivery for local poor and underserved populations
4. Clinical opportunity with the Department of Public Health

**Q18** Please rank the barriers in developing global health training.

1. Time
2. Money
3. Malpractice Insurance
4. Language barriers
5. Political conflict
6. Lack of Mentorship
7. No Interest

**Q19** Any additional comments or questions?
